# Geographic Pattern of Sushi Product Misdescription in Italy—A Crosstalk between Citizen Science and DNA Barcoding

**DOI:** 10.3390/foods10040756

**Published:** 2021-04-02

**Authors:** Anna Maria Pappalardo, Alessandra Raffa, Giada Santa Calogero, Venera Ferrito

**Affiliations:** Department of Biological, Geological and Environmental Sciences, Section of Animal Biology “M. La Greca”, University of Catania, Via Androne 81, 95124 Catania, Italy; alessandra.raffa92@gmail.com (A.R.); giadacalogero@gmail.com (G.S.C.); vferrito@unict.it (V.F.)

**Keywords:** sushi restaurants, COI barcoding, molecular traceability, teleosts

## Abstract

The food safety of sushi and the health of consumers are currently of high concern for food safety agencies across the world due to the globally widespread consumption of these products. The microbiological and toxicological risks derived from the consumption of raw fish and seafood have been highlighted worldwide, while the practice of species substitution in sushi products has attracted the interest of researchers more than food safety agencies. In this study, samples of sushi were processed for species authentication using the Cytochrome Oxidase I (COI) gene as a DNA barcode. The approach of Citizen Science was used to obtain the sushi samples by involving people from eighteen different Italian cities (Northern, Central and Southern Italy). The results indicate that a considerable rate of species substitution exists with a percentage of misdescription ranging from 31.8% in Northern Italy to 40% in Central Italy. The species most affected by replacement was *Thunnus thynnus* followed by the flying fish roe substituted by eggs of *Mallotus villosus*. These results indicate that a standardization of fish market names should be realized at the international level and that the indication of the scientific names of species should be mandatory for all products of the seafood supply chain.

## 1. Introduction

In part I of Food Business Regulation (Cap. 132X) of the Government of the Hong Kong Special Administrative Region, the meanings of the terms sushi and sashimi are made explicit. In particular, sushi is described as “food consisting of cooked and pressed rice flavoured with vinegar and garnished with other food ingredients including raw or cooked or vinegared seafood, marine fish or shellfish roe, vegetable, cooked meat or egg on top or in the middle which may or may not be wrapped with seaweed and usually served in pieces”, while sashimi is described as “food consisting of fillets of marine fish, molluscs, crustaceans, fish roe or other seafood to be eaten in raw state”. Although sushi and sashimi are perceived by consumers as healthy foods, the biological and chemical hazards for human health, derived from the consumption of raw fish and seafood, have been highlighted worldwide, such as the risk of parasitic and/or pathogenic microorganism infection [1,2,3,4,5,6,7]; the potential risk arising from a lack of proper control of temperature of these perishable foods [8]; the risk of exposure to toxicants, such as heavy metals and polychlorinated biphenyls (PCBs); polycyclic aromatic hydrocarbons (PAHs) and other contaminants [4,7,9,10]. The food safety of sushi and sashimi and the health of consumers are currently of high concern given that the consumption of these products is now globally widespread [11,12]. As a result, the most important food safety agencies in the world, such as the European Food Safety Authority [13], the Food and Drug Administration [14], the Hong Kong Food and Environmental Hygiene Department’s and the World Health Organization, have implemented regulations and guidelines to face all issue related to the consumption of raw fish and seafood. In this context, another important issue that has attracted the interest of researchers is the molecular authentication of fish and seafood species in transformed products, because the processing procedure generally removes the specific diagnostic morphological traits useful to assign the product to a particular species through only morphological inspection. Indeed, the voluntary or involuntary practices of substitution of valuable species with species of less value for economic profit have been detected worldwide in the last decades by using DNA sequencing, which proved to be the most useful method to unveil these frauds. For example, DNA-based surveys carried out in European and non-European countries have highlighted a high rate of food frauds in the fishery sector [15,16,17,18]. Among the most used molecular markers, mitochondrial genes, such as Cytochrome b (Cytb), 16S rRNA (16S), Cytochrome Oxidase I (COI) and mtDNA Control Region (CR), have proven to be optimal tools for seafood species authentication. However, while the CR and Cytb have been successfully and widely used to study the genetic population structure [19,20,21,22,23,24,25,26] rather than to authenticate fish species [27,28,29], COI has become the optimal DNA barcode for the identification of animal species [30,31,32,33,34,35] and particularly for fish species authentication in seafood products [15,36,37,38,39,40,41]. Furthermore, researchers have been also encouraged to look for rapid and low-cost molecular strategies to tackle substitution species frauds by large scale screening both using classic and new technologies [42,43,44,45,46,47,48,49]. COI DNA barcoding has been used to unveil the misdescription of sushi products in the United States of America [50], the United Kingdom [11], South-Korea [17], Malaysia [51,52] and Canada [53]. In Italy, the study by Armani et al. [54] performed a molecular-based authentication of the seafood species used in sushi preparations in four provinces of Tuscany. However, when designing a food fraud investigation, the sampling plan is pivotal to ensure that as many products as possible are sampled over a large area. In this context, the contribution of consumers is crucial, and the citizen science (CS) approach, based on involving a large number of people, normally including the local population of a region or a state, with the aim of collecting scientific data, could prove to be of fundamental help. This strategy permits the collection of a vast quantity of data information or samples that cannot be collected by only one researcher or a small research team. The quality of a study is not undermined by the citizen science approach if the work planning includes comprehensible protocols, effective training before starting and accurate oversight during the studying period [55,56]. Based on these premises, in this study, we involved many people from eighteen different cities throughout the Italian territory (North, Central and South Italy) to obtain samples of sushi to be processed for species authentication by using the COI gene as the DNA barcode. To the best of our knowledge, this is the first study on sushi authentication extended to the Italian territory by using the approach of citizen sciences. The aim was to analyze the compliance of the fish names of marketed products with the list of Italian names of fish species of commercial interest included in the Italian Ministerial Decree (MD) n.19105 22 September 2017 of the Italian Ministry of Agricultural, Food and Forestry Policies and then to verify if the information the consumers obtain from the menu meet the transparency requirements established by the European regulations.

## 2. Materials and Methods

### 2.1. Sample Collection and Survey

**Figure 1:**
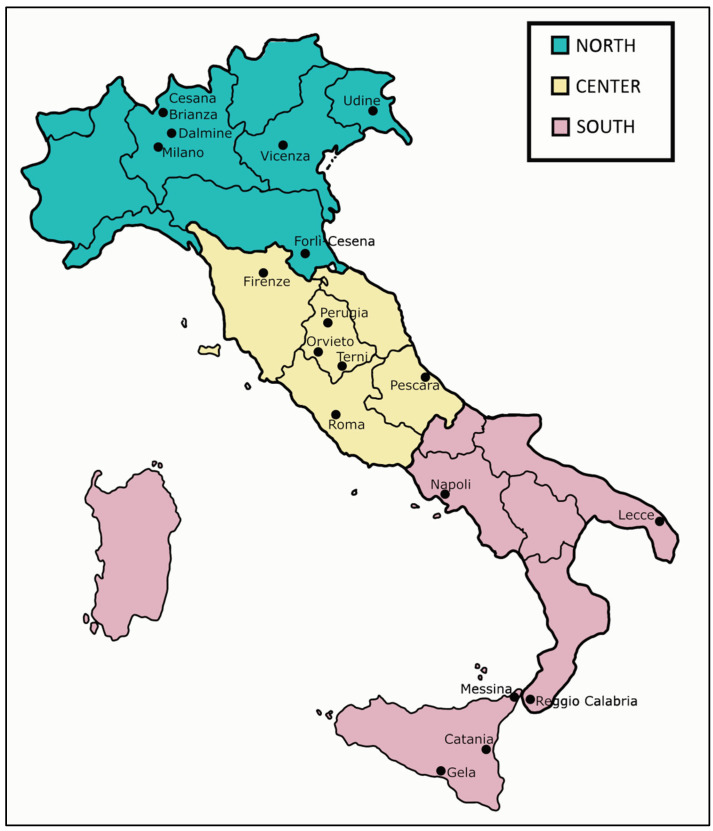
Collection sites of the sushi survey in Northern (green), Central (light yellow) and Southern (pink) Italy.

Between January 2018 and January 2019, we collected sushi products sold in restaurants and takeaways in different cities of Northern, Central and Southern Italy (Figure 1). Samples were obtained using a “citizen science” strategy involving people who responded to the invitation to participate in the “sushi survey”. People living in various Italian regions were chosen among relatives and friends of our research team and colleagues at the University of Catania. This allowed us to establish a direct contact with them to better program the sampling. Prior to the start of the study, people received a letter from us where we explained our research project and asked them about their willingness to participate. After receiving their consent, we contacted them by phone and also via skype (i) to respond to all queries they would ask us; (ii) to explain how to proceed for sampling sushi products; and (iii) how to fill in the documents that they would receive by us. In particular, we advised them to focus the sampling on white fish, tuna and eggs. By mail, we provided participants with a step-by-step guide for sampling, including a sample collection table (Figure S1: sushi sampling guide) together with a 1.5 mL eppendorf tube to be used to preserve small pieces of sampled sushi in 95% ethanol. A stamped envelope to be used to send us the samples and the collection table was also included. In the table, participants indicated the sushi venue (restaurant or takeaway) they visited; the name on the menu of the product they consumed; and how many samples among white fish, tuna and/or eggs they collected.

### 2.2. DNA Barcoding Analysis

A total of 180 samples were processed for DNA analysis. For each sushi product, 3 DNA extractions were replicated to investigate the presence of multiple fish species in the product. Total genomic DNA was extracted using a DNeasy tissue kit (Qiagen, Hilden, Germany) following the manufacturer’s instructions and with some modifications. DNA concentration was measured with a NanoDrop One spectrophotometer (Thermo Scientific, Waltham, MA, USA). A portion of about 650 bases of the COI gene was amplified following the Polymerase Chain Reaction (PCR) conditions reported by [38] in a 50 µL reaction mixture also containing the M13 tailed primers (VF2_t1 and FishR2_t1) described in Ivanova et al. [57] to improve the sequencing quality of the PCR products. Negative controls were included in all PCR runs to check for cross-contamination. Amplicons successfully obtained were verified by electrophoresis on a 0.8% agarose gel and displayed through a Safe Imager TM 2.0 Blue Light Transilluminator (Thermo Fisher, Waltham, MA, USA) using the SYBR^®^ Safe dye (Thermo Fisher, Waltham, MA USA). The QIAquick PCR purification kit (Qiagen, Hilden, Germany) was used to purify all amplicons, which were then bidirectionally sequenced with M13 sequencing primers using an ABI 3730 automated sequencing machine at Genechron Biotech Company (https://www.genechron.com accessed on 30 January 2021).

### 2.3. Data Analysis

The chromatograms were checked for the quality of peaks and assembled using ChromasPro 2.6.6 software (https://technelysium.com.au/wp/chromaspro/ accessed on 30 January 2021). Barcode multiple-sequence alignment was carried out using the online version of MAFFT v.7 [58]. Sequences were trimmed when the errors occurred near the beginning and again at the end of any sequence. Primer sequences were manually removed by using BioEdit 7.2 (https://bioedit.software.informer.com/versions/ accessed on 30 January 2021). The obtained sequences were carefully checked for the presence of nuclear mitochondrial pseudogenes or nuclear mitochondrial DNA sequences (NUMTs), which could be easily coamplified with orthologous mtDNA sequences [59]. The translation of nucleotide sequences to amino acids was performed by the EMBOSS Transeq tool (https://www.ebi.ac.uk/Tools/st/emboss_transeq accessed on 30 January 2021January) in order to check for premature stop codons and to verify that the open reading frames were maintained in the protein-coding locus. To confirm the identity of the amplified sequences, we conducted Basic Local Alignment Searches (BLAST) (https://blast.ncbi.nlm.nih.gov accessed on 30 January 2021) against GenBank without “Uncultured/environmental sample sequences” with megablast and default parameters (https://www.ncbi.nlm.nih.gov/genbank/ accessed on 30 January 2021) and also used the BOLD database (https://www.boldsystems.org/ accessed on 30 January 2021) to validate our sequences. For species assignment, the highest values of percent identity found between the query sequence and the BLAST matched sequences were selected. If multiple BLAST matches had identical percent identity values, it was confirmed that all matches belonged to the same species. All sequences obtained from the present study were published in the National Center for Biotechnology Information database (NCBI), and their GenBank accession numbers are reported in Table 1, Table 2 and Table 3.

## 3. Results

### 3.1. Sampling

A total of 61 sushi samples consisting of 45 fish samples, white fish and tuna, and 16 roe samples were collected from 15 restaurants and 14 takeaways from people living in 18 Italian cities who responded to the invitation to participate in the “sushi survey” (Figure 1). For each sushi venue, participants collected from 1 to 3 samples; in the latter case, “white fish”, “tuna” and “fish roe” were sampled. The initial instructions provided by us to the participants in the survey allowed us to obtain a homogeneous, high-quality sampling plan throughout the territory. In Table 1, Table 2 and Table 3, the names found on the menu/label for each sample were reported, as well the corresponding scientific names of the declared species found in the list of the Italian names of fish species of commercial interest included in the Italian ministerial decree (MD) 21 September 2017. Misdescription was marked up when no match was found among the name on the menu, the scientific name in the list of the MD and the fish species identified by DNA barcoding.

### 3.2. DNA Barcoding

Three samples of each sushi product for a total of 180 samples were processed; however, DNA extraction was unsuccessful for 17 samples, and a total of 163 COI DNA sequences were obtained. The presence of multiple fish species was not detected after the COI sequencing of three samples for each examined product. The sequence length was between 636 and 655 bp. In these functional mitochondrial COI sequences, no insertions, deletions or stop codons were observed, and NUMTs were not sequenced given that vertebrate NUMTS are generally smaller than 600 bp [59]. A total of 16 fish species were identified in all examined sushi products. The percent identity between the COI query sequences and their top-match sequences ranged from 98.17 to 99.85 with 100% of sequence coverage (Table 1, Table 2 and Table 3).

### 3.3. Geographic Pattern of Sushi Product Misdescription

#### 3.3.1. Northern Italy

Red tuna, *Thunnus thynnus*, was substituted by yellowfin tuna, *T. albacares*, in three cases and by bigeye tuna, *T. obesus*, in one case; sea bream, *Sparus aurata*, was substituted in one case by yellowtail amberjack, *Seriola lalandi*. Concerning fish roe, only in one case, under the name tobiko or flying fish roe, the eggs of *Mallotus villosus* were found in place of the eggs of species of the genus *Hirundichthys* (Table 1).

#### 3.3.2. Central Italy

In five cases, *T. thynnus* was substituted by *T. albacares* and in one case by *T. orientalis*, while tobiko or flying fish eggs were substituted by *M. villosus* eggs (Table 2).

#### 3.3.3. Southern Italy

In all cases, red tuna, *T. thynnus*, was substituted by *T. albacares*. Sea bream was substituted in one case by *Xiphias gladius* and in another case by the bluefish, *Pomatomus saltatrix*. Tobiko or flying fish eggs in one case were substituted by the eggs of *M. villosus*.

Based on the names of the products chosen by consumers on the menu, a total of 17 species should have been detected, but we found a total of 29 species (Table 3).

## 4. Discussion

The results of the survey on the authentication of fish species used for sushi products sold in restaurants and takeaways in Italy indicate that a considerable rate of species substitution exists throughout the territory and that it is focused on certain species. The percentage of misdescription ranges from 31.8% in Northern Italy to 40% in Central Italy. The rate of misdescription affecting takeaways ranges from 25% of cases in Northern Italy to 50% in Southern Italy, while the percentage of misdescription in restaurants ranges from 33.3% in Southern Italy to 50% in Central Italy. The species most affected by replacement was *Thunnus thynnus*, which was substituted in 67% of cases in Northern Italy and 100% of cases in Central and Southern Italy. The so-called “white fish” usually represented by *S. aurata* and *D. labrax* was affected by a low rate of substitution ranging from 11% in Northern Italy to 22% in Southern Italy. Finally, tobiko or flying fish roe was affected by a medium rate of substitution ranging from 20% in Central Italy to 33% in Northern Italy. Before discussing our results, it should be noted that i) we compared them with those obtained from a similar survey carried out in Italy and in European and non-European countries, and ii) the cases of misdescription detected in the present study were based on the incongruence found between the scientific or common names of the species declared on the menu at the retailers (sushi restaurant and takeaway), the specific molecular diagnosis obtained through the COI DNA barcoding and the corresponding denomination in Italian language to be attributed to the detected fish species, as indicated in the decree of the Ministry of Agricultural, Food and Forestry Policies (MD n. 19105 22 September 2017) dealing with the Italian names of fishes of commercial interest. In particular, the MD clearly states that to correctly inform consumers, the name to be used to indicate *T. thynnus* is “tuna” or “red tuna”, while the name “yellowfin tuna” must be used to indicate *T. albacares*, and the names, “orientalis or oceanic tuna” and “bigeye tuna”, should be used to indicate the species *T. orientalis* and *T. obesus,* respectively. Based on this premise, the high percentage of misdescription found for *T. thynnus* is shown by the fact that only in two cases out of 16, consumers really ate red tuna as declared on the menu, while in 87.5% of cases, they consumed yellowfin tuna (12 cases), orientalis tuna (1 case) and bigeye tuna (1 case) in place of red tuna. The survey carried out in Italy by Armani et al. [54] on misdescription in sushi products sold in Tuscany revealed a generally low rate of misdescription (3.4%), which in any case did not concern tuna-based products. However, the authors identified the products sold as tuna only at the genus level and then as belonging to the genus *Thunnus*, because EU regulations (1379/2013 and 1169/2011) require only the name of the seafood category and not the name of the species at the catering level. Similarly, a moderate level of species substitution (10%) was detected by Vandamme et al. [11] during a screening of seafood labelling accuracy in sushi bars and restaurants across England. The low rate of substitution detected for tuna products was imputed to the United Kingdom labelling regulations allowing the inclusion of all *Thunnus* species under the umbrella term “tuna’’. Interestingly, high levels of mislabeling (83.3%) for Bluefin tuna, *T. thynnus*, like those detected by us, were detected in French sushi restaurants, compared with the low general substitution rate (3.6%) observed over the whole sampling [15]. An intermediate level of species substitution was detected by Oceana [60] in a survey carried out in sushi restaurants in Brussels, where a 54.5% level of fraud was found, mainly due to the frequent substitution of *T. thynnus* by others cheaper tropical tuna species (*T. albacares* and *T. obesus*). Both in the United States of America and in China, the species of the genus *Thunnus* are sold under the umbrella terms ‘‘tuna’’ according to the Food and Drug Administration and the Food and Drugs (Composition and Labelling) Regulations (Cap. 132W), respectively [50,61]. However, the molecular screening carried out by Lowenstein et al. [50] in the United States of America led to the identification of sushi tuna samples up to the level of species by highlighting the substitution of bluefin tuna by different species in 40% of samples. A case of the substitution of *T. obesus* by *T. thinnus* has also been observed in sushi products in Canada, which could raise suspicion of illegal, unreported and unregulated fishing issue [53]. Instead, the investigation carried out by However et al. [61] in Honk Kong stated that tuna samples, identified only at the genus level, were correctly labeled.

Focusing our attention on the other cases of species substitution observed in our study, three species, *S. lalandi* (Yellowtail amberjack named oceanic amberjack in the Italian list of the species), *X. gladius* (swordfish) and *P. saltatrix* (bluefish), were found in place of *S. aurata* declared on the menu. In this case, there is no doubt that the species substitution was deliberate, although the economic profit may not be the incentive to defraud, but rather the ease of finding the species. The Yellowtail amberjack is an aquaculture species often consumed as sashimi reared in Japan, Australia and New Zealand. In recent decades, the bluefish has undergone a rapid northern range expansion within the Mediterranean from the southern and eastern sectors of the basin. This geographical expansion has been demonstrated to be a result of increasing water temperature [62] and is having an important socio-economic impact due to the voracious behavior of this predator [63]. However, the presence of *X. gladius* in place of *S. aurata* is of major concern, as swordfish is a species of greater economic value than seabream, and in this case, substitution could launder illegally caught swordfish. Another frequent case of species substitution observed by us was the substitution of flying-fish eggs or tobiko by eggs of capelin, *M. villosus*. Flying fish are all included within the family Exocoetidae, and the term tobiko indicates the roe of flying fish of the genera *Cheilopogon* and *Hyrundichthys* generally used in sushi preparation. Tobiko is made of small eggs of 2 mm or less in size, which are crisp and of golden orange color. Due to the small supply of flying fish roe, tobiko are often prepared by using immature roe of capelin or other fish which might be also colored and sold as imitation [64,65]. The Italian MD n. 19105 22 September 2017 includes the names of only two taxa of flying fish: the “oceanic flying-fish”, which is an umbrella name for the species of *Cypselurus* spp., and “Indopacific flying-fish”, which is used to indicate the species *Cheilopogon atrisignis*. Therefore, we considered only the above cases of substitution concerning *M. villosus* as misdescriptions, which was also reported by Armani et al. [54] in Tuscany and by Wallstrom et al. [66] in sushi bars in Honolulu. The results obtained from the molecular survey carried out in Italy indicate the effectiveness of COI barcoding for fish authentication in sushi products and highlight two main issues: (i) it is evident that a revision of the regulations by making the use of the scientific names of species mandatory for all products of the seafood supply chain is the only way to protect consumers from frauds, to guarantee their health, to protect the threatened species from illegal fishing and to restore the depleted fish stocks; (ii) to achieve these goals, a standardization of fish market names, avoiding using the same trade name to indicate multiple species, should be realized at the international level given that the fish market is now globalized.

Finally, the results of our study were obtained using the approach of Citizen Science, which allowed us to cover a wide portion of the Italian territory for the sushi survey. This relatively new approach was used by Bernard-Capelle et al. [15] to detect the rate of fish mislabeling in France and by Pardo et al. [67] to carry out a survey on seafood mislabeling in restaurants of 23 states across Europe. The most important benefit for researchers engaging citizens to obtain information for scientific investigations is the possibility to collect a high number of samples covering a wide geographical area controlling costs resulting from sampling. On the other hand, citizens, as consumers, will become aware of food safety concerns, which could be difficult to perceive by the end users of the food chain.

## Figures and Tables

**Table 1:** Sushi sampling in Northern Italy. In square brackets, the number of processed samples for each sushi product. In bold, misdescription cases.

Code *	Retail Point	Menu/Label Description	Scientific Nameof Declared Species	Identified Species by DNA Barcoding and BLAST Search	GenBank Acc. Number of Obtained Sequences	Matched GenBank Accession from BLAST °	Matched BOLD ID	% Identity with 100% Coverage
MIL1B [3]	Restaurant	sea bream or common bass	*Sparus aurata/Dicentrarchus labrax*	*Dicentrarchus labrax*	MW714726	KP330301	GBMIN94166-17	99.69
MIL1T [3]	Restaurant	tuna	*Thunnus thynnus*	***Thunnus albacares***	MW714727	MH638785	ANGBF54814-19	99.54
MIL2B [1]	Takeaway	sea bream or common bass	*Sparus aurata/Dicentrarchus labrax*	*Dicentrarchus labrax*	MW714728	KY176457	GBMIN121550-17	99.37
MIL2T [3]	Takeaway	tuna	*Thunnus thynnus*	***Thunnus albacares***	MW714729	MH638777	ANGBF54806-19	98.47
MIL3B [3]	Takeaway	sea bream	*Sparus aurata/Dicentrarchus labrax*	*Dicentrarchus labrax*	MW714730	KP330301	GBMIN94165-17	98.74
MIL4B [3]	Takeaway	common bass	*Sparus aurata/Dicentrarchus labrax*	*Sparus aurata*	MW714731	MF438138	ANGBF45411-19	99.54
CES1B [3]	Takeaway	common bass	*Dicentrarchus labrax*	*Dicentrarchus labrax*	MW714732	KP330300	GBMIN94165-17	99.06
DAL1B [3]	Restaurant	sea bream	*Sparus aurata*	*Sparus aurata*	MW714733	JQ623999	DNATR096-12	99.24
DAL1T [3]	Restaurant	tuna	*Thunnus thynnus*	***Thunnus albacares***	MW714734	MH638785	ANGBF54814-19	99.24
DAL1E [3]	Restaurant	tobiko/flying fish egg	*Hirundichthys affinis °*	*Hirundichthys oxycephalus*	MW714735	KX769042	GBMIN125981-17	99.02
VI1T [3]	Restaurant	tuna	*Thunnus thynnus*	*Thunnus thynnus*	MW714736	KP975912	FCSF387-14	98.92
VI1B [3]	Restaurant	sea bream	*Sparus aurata*	*Sparus aurata*	MW714737	KC501553	DNATR1582-13	98.78
VI1E [3]	Restaurant	tobiko/flying fish egg	*Hirundichthys affinis °*	*Hirundichthys affinis*	MW714738	JQ842898	TOBA086-09	99.52
FC1B [3]	Takeaway	sea bream	*Sparus aurata*	***Seriola lalandi***	MW714739	MH211123	GBMNA18700-19	99.39
FC1T [3]	Takeaway	maguro Yaki (red tuna)	*Thunnus thynnus*	*Thunnus thynnus*	MW714740	KC501694	DNATR1720-13	99.39
FC2B [1]	Takeaway	sea bream	*Sparus aurata*	*Sparus aurata*	MW714741	MF438138	ANGBF45411-19	99.69
UD1B [3]	Restaurant	kajiki roll (swordfish)	*Xiphias gladius*	*Xiphias gladius*	MW714742	MK295657	ANGBF51916-19	99.38
UD1T [2]	Restaurant	tuna	*Thunnus thynnus*	***Thunnus obesus***	MW714743	GU451774	GBGCA1353-13	99.08
UD1E [3]	Restaurant	tobiko/flying fish egg	*Hirundichthys affinis °*	***Mallotus villosus***	MW714744	HM421773	DSFAL635-09	99.39

* MIL = Milano; CES = Cesena Brianza; DAL = Dalmine; VI = Vicenza; FC = Forlì Cesena; UD = Udine. ° These species are not present in the Italian D.M. 2008.

**Table 2:** Sushi sampling in Central Italy. In square brackets, the number of processed samples for each sushi product. In bold, misdescription cases.

Code *	Retail Point	Menu/Label Description	Scientific Name of Declared Species	Identified Species by DNA Barcoding and BLAST Search	GenBank Acc. Number of Obtained Sequences	Matched GenBank Accession from BLAST °	Matched BOLD ID	% Identity with 100% Coverage
FIR1B [3]	Takeaway	common bass	*Dicentrarchus labrax*	*Dicentrarchus labrax*	MW714657	KP330300	GBMIN94165-17	99.21
FIR1T [3]	Takeaway	tuna	*Thunnus thynnus*	***Thunnus albacares***	MW714658	MH638777	ANGBF54806-19	99.85
FIR1E [3]	Takeaway	tobiko/flying fish egg	*Hirundichthys affinis °*	*Hirundichthys affinis*	MW714659	JQ842898	TOBA9086	99.52
PER1B [3]	Restaurant	sea bream or common bass	*Sparus aurata/Dicentrarchus labrax*	*Sparus aurata*	MW714660	MF438138	ANGBF45411-19	99.54
PER1T [3]	Restaurant	tuna	*Thunnus thynnus*	***Thunnus albacares***	MW714661	MH638785	ANGBF54814-19	99.39
PER1E [1]	Restaurant	tobiko/flying fish egg	*Hirundichthys affinis °*	*Hirundichthys affinis*	MW714662	JQ842898	TOBA9086	99.35
PER2B [3]	Takeaway	sea bream or common bass	*Sparus aurata/Dicentrarchus labrax*	*Dicentrarchus labrax*	MW714663	KP330301	GBMIN94166-17	99.21
ORV1B [3]	Takeaway	common bass	*Dicentrarchus labrax*	*Dicentrarchus labrax*	MW714664	KP330301	GBMIN94166-17	98.58
ORV1T [2]	Takeaway	tuna	*Thunnus thynnus*	***Thunnus albacares***	MW714665	MH638785	ANGBF54814-19	98.17
ORV1E [3]	Takeaway	tobiko/flying fish egg	*Hirundichthys affinis °*	*Hirundichthys affinis*	MW714666	JQ842898	TOBA086-09	99.52
ORV2B [3]	Restaurant	sea bream	*Sparus aurata*	*Sparus aurata*	MW714667	KC501553	DNATR1582-13	98.47
TER1B [3]	Takeaway	sea bream	*Sparus aurata*	*Sparus aurata*	MW714668	KC501557	DNATR1596-13	99.39
TER1T [3]	Takeaway	tuna	*Thunnus thynnus*	***Thunnus orientalis***	MW714669	JN097817	GBGCA1390-13	99.70
TER1E [3]	Takeaway	tobiko/flying fish egg	*Hirundichthys affinis °*	*Hirundichthys affinis*	MW714670	JQ842898	TOBA9086	99.52
PE1B [3]	Restaurant	common bass	*Dicentrarchus labrax*	*Dicentrarchus labrax*	MW714671	KP330301	GBMIN94166-17	98.58
PE1T [3]	Restaurant	tuna	*Thunnus thynnus*	***Thunnus albacares***	MW714672	MH638785	ANGBF54814-19	98.92
PE1E [1]	Restaurant	tobiko/flying fish egg	*Hirundichthys affinis °*	*Hirundichthys coromandelensis*	MW714673	KX379460	ANGBF32076-19	98.73
RO1B [3]	Restaurant	sea bream	*Sparus aurata*	***Seriola lalandi***	MW714674	MF069453	ANGBF17684-19	99.24
RO1T [3]	Restaurant	tuna	*Thunnus thynnus*	***Thunnus albacares***	MW714675	HM007768	ANGBF7098-12	99.39
RO1E [1]	Restaurant	tobiko/flying fish egg	*Hirundichthys affinis °*	***Mallotus villosus***	MW714676	FJ205579	GBGC7486-09	99.23

* FIR = Firenze; PER = Perugia; ORV = Orvieto; TER = Terni; PE = Pescara; RO = Roma. ° These species are not present in the Italian D.M. 2008.

**Table 3:** Sushi sampling in Southern Italy. In square brackets, the number of processed samples for each sushi product. In bold, misdescription cases.

Code *	Retail Point	Menu/Label Description	Scientific Name of Declared Species	Identified Species by DNA Barcoding and BLAST Search	GenBank Acc. Number of Obtained Sequences	Matched GenBank Accession from BLAST °	Matched BOLD ID	% Identity with 100% Coverage
CAT1B [3]	Restaurant	sea bream or common bass	*Sparus aurata*	*Sparus aurata*	MW714949	KC501553	DNATR1582-13	99.08
CAT1T [3]	Restaurant	Tuna	*Thunnus thynnus*	***Thunnus albacares***	MW714950	MH638785	ANGBF54814-19	99.54
CAT1E [3]	Restaurant	tobiko/flying fish egg	*Hirundichthys affinis °*	***Mallotus villosus***	MW714951	FJ205579	GBGC7486-09	99.39
CAT3B [3]	Restaurant	sea bream or common bass	*Sparus aurata*	***Xiphias gladius***	MW714952	JN049558	ANGBF7251-12	99.54
GE1B [3]	Restaurant	common bass	*Dicentrarchus labrax*	*Dicentrarchus labrax*	MW714953	KP330301	GBMIN94166-17	99.53
GE1T [3]	Restaurant	Tuna	*Thunnus thynnus*	***Thunnus albacares***	MW714954	MH638785	ANGBF54814-19	99.08
GE1E [3]	Restaurant	tobiko/flying fish egg	*Hirundichthys affinis °*	*Hirundichthys affinis*	MW714955	JQ842898	TOBA086-09	99.52
GE2B [3]	Restaurant	common bass	*Dicentrarchus labrax*	*Dicentrarchus labrax*	MW714956	KP330301	GBMIN94166-17	99.53
ME1B [3]	Restaurant	sea bream	*Sparus aurata*	*Sparus aurata*	MW714957	MF438138	ANGBF45411-19	99.85
ME1T [3]	Restaurant	Tuna	*Thunnus thynnus*	***Thunnus albacares***	MW714958	MH638762	ANGBF54791-19	98.93
ME1E [3]	Restaurant	lumpfish roe	*Cyclopterus lumpus*	*Cyclopterus lumpus*	MW714959	MG421634	TZAIC166-05	99.54
ME2B [3]	Restaurant	sea bream	*Sparus aurata*	*Sparus aurata*	MW714960	MF438138	ANGBF45411-19	99.39
ME2E [2]	Restaurant	Ikura	salmon eggs	*Oncorhynchus keta*	MW714961	LC094477	ANGBF41103-19	98.93
RC1B [3]	Takeaway	Anago	*Anguilla sp*	*Anguilla rostrata*	MW714962	KX459333	SERCA165-12	98.31
RC1T [3]	Takeaway	Tuna	*Thunnus thynnus*	***Thunnus albacares***	MW714963	MH638785	ANGBF54814-19	99.39
RC1E [2]	Takeaway	Ikura	salmon eggs	*Oncorhynchus keta*	MW714964	LC094477	ANGBF41103-19	99.54
RC2E [2]	Takeaway	lumpfish roe	*Cyclopterus lumpus*	*Cyclopterus lumpus*	MW714965	MG421634	TZAIC166-05	99.07
LE1B [3]	Restaurant	sea bream	*Sparus aurata*	*Sparus aurata*	MW714966	MF438138	ANGBF45411-19	98.93
LE1E [1]	Restaurant	tobiko/flying fish egg	*Hirundichthys affinis °*	***Mallotus villosus***	MW714967	FJ205579	GBGC7486-09	99.39
NA1B [3]	Takeaway	sea bream or common bass	*Sparus aurata/Dicentrarchus labrax*	*Sparus aurata*	MW714968	KC501554	DNATR1599-13	99.24
NA2B [3]	Takeaway	sea bream or common bass	*Sparus aurata/Dicentrarchus labrax*	***Pomatomus saltatrix***	MW714969	KC501113	DNATR1143-13	99.39

* NA = Napoli; LE = Lecce; RC = Reggio Calabria; ME = Messina; CAT = Catania; GE = Gela. ° These species are not present in the Italian D.M. 2008.

## Supplementary Materials

The following are available online at https://www.mdpi.com/article/10.3390/foods10040756/s1, Figure S1: sushi sampling guide.
