# Supplementary material for: Geographic Pattern of Sushi Product Misdescription in Italy—A Crosstalk between Citizen Science and DNA Barcoding
Source: Foods. 2021 Apr 2;10(4):756. doi: 10.3390/foods10040756 (PMC8066630; doi:10.3390/foods10040756)
Supplement: Supplementary file 1 [file foods-10-00756-s001.pdf]

SUSHI SAMPLING GUIDE

DATE __/__/____

Sampler's name: ___________________________ City: ________________________________

The kit includes:

- Presentation letter.
- Steril Eppendorf numbered from 1 to 4, containing 96% ethanol.
- Sterile disposable scalpels and tweezers and Parafilm;
- Sampling datasheet (see below) to be filled in and sent together with samples
- Stamped envelope for the shipment of samples and datasheet

Recommendation before starting the sampling

- Wash the hands;
- Clean the work surface or use aluminum foil.
- Avoid the contamination of the sample.
- If possible, take pictures of menù, dishes, and ingredients.
- The sample must be taken from a part not in contact with rice, seaweed, or other seafood types.

SECTION TO BE FILLED IN

Indicate the sushi venue (put an X on the square):

- ☐ Ristorante
- ☐ Take away

Write below every numbered tube the sample type (WHITE FISH, TUNA, EGGS) and report the species indicated in the menù (see the example).

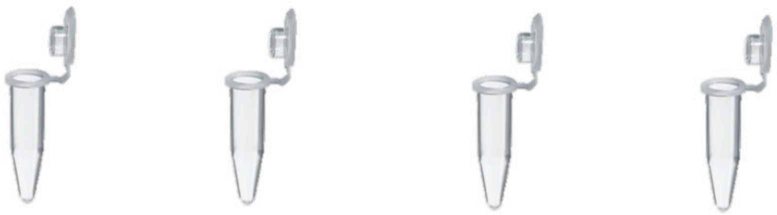

1 2 3 4

Example:

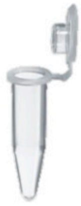

1

____________ ______________ _______________ ______________ *White fish -Seabass*

Finally, envelope each tube with a Parafilm piece (included in the kit) to seal the caps and prevent their opening during transport. Put the tubes and this filled datasheet in the stamped envelope and sent it.
